# Structures of Bacterial RNA Polymerase Complexes Reveal the Mechanism of DNA Loading and Transcription Initiation

**DOI:** 10.1016/j.molcel.2018.05.021

**Published:** 2018-06-21

**Authors:** Robert Glyde, Fuzhou Ye, Milija Jovanovic, Ioly Kotta-Loizou, Martin Buck, Xiaodong Zhang

**Affiliations:** 1 Section of Structural Biology, Department of Medicine, Imperial College London, London SW7 2AZ, UK; 2 Department of Life Sciences, Imperial College London, London SW7 2AZ, UK

**Keywords:** transcription initiation, DNA opening, transcription bubble, RNA polymerase, sigma, *de novo* RNA synthesis, protein-DNA interactions, promoter DNA, transcription activation, bacterial enhancer-binding proteins

## Abstract

Gene transcription is carried out by multi-subunit RNA polymerases (RNAPs). Transcription initiation is a dynamic multi-step process that involves the opening of the double-stranded DNA to form a transcription bubble and delivery of the template strand deep into the RNAP for RNA synthesis. Applying cryoelectron microscopy to a unique transcription system using σ^54^ (σ^N^), the major bacterial variant sigma factor, we capture a new intermediate state at 4.1 Å where promoter DNA is caught at the entrance of the RNAP cleft. Combining with new structures of the open promoter complex and an initial *de novo* transcribing complex at 3.4 and 3.7 Å, respectively, our studies reveal the dynamics of DNA loading and mechanism of transcription bubble stabilization that involves coordinated, large-scale conformational changes of the universally conserved features within RNAP and DNA. In addition, our studies reveal a novel mechanism of strand separation by σ^54^.

## Highlights

• Structure of an intermediate transcription initiation complex
• Structures of an open complex and a *de novo* initial transcribing complex
• A coupled DNA load and unwind model for transcription initiation
• Transcription bubble stabilized by a helix separating the two DNA strands

## Graphical Abstract

**Figure:**
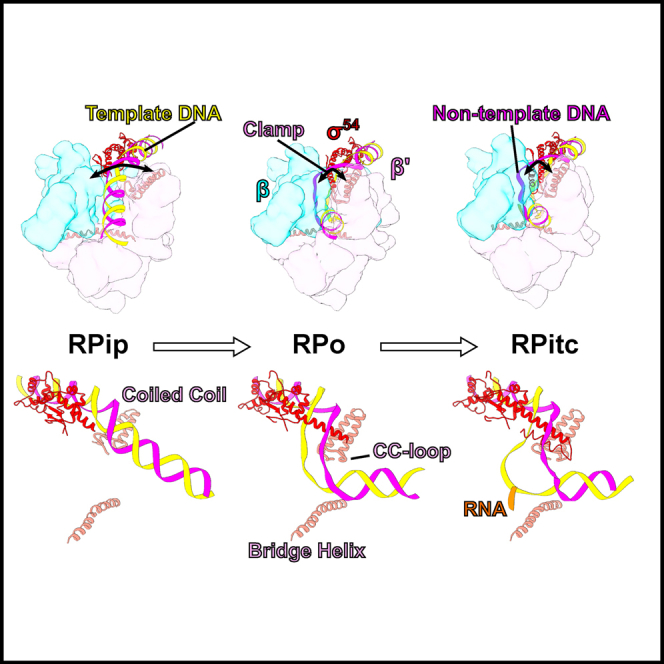

## Teaser

Glyde et al. report three cryo-EM structures of bacterial transcription initiation complexes. These structures support a coupled DNA load and unwind model that involves an initial opening of the RNA polymerase clamp to facilitate DNA loading before it closes down to complete the loading, which also leads to DNA unwinding.

## Introduction

Gene transcription is a fundamental cellular process carried out by the multi-subunit RNA polymerase (RNAP), which is conserved from bacteria to humans (Cramer, 2002, Werner, 2008). Transcription consists of a number of key stages including the recruitment of RNAP to the promoter site, initiation, initial RNA synthesis, elongation, and termination. Significant advances have been made in recent years in determining how RNAPs are recruited as well as how they synthesize RNA (Blombach et al., 2016, Hantsche and Cramer, 2016, Liu et al., 2013, Murakami, 2015). However, the initiation process, which involves the opening up ∼12 base pairs of the initially double-stranded promoter DNA (dsDNA) and delivery of the template (T) strand DNA into the RNAP active site, is still poorly understood, partly due to the dynamic and transient nature of key complexes. General transcription factors and sigma factors are required to recruit RNAP to promoter sites in eukaryotes and bacteria, respectively (Blombach et al., 2016, Gruber and Gross, 2003). In bacteria, σ^70^ controls housekeeping genes and is the archetypical sigma factor of its class. σ^70^ recruits RNAP to promoter sites by recognizing −10 and −35 consensus sequences (upstream relative to the transcription start site [TSS] at +1) and forms a closed complex (RPc) that can then spontaneously isomerize to an open complex (RPo). The recruitment and isomerization processes are thus coupled and the RPc has not yet been defined structurally for a σ^70^ system (Browning and Busby, 2016, Feklístov et al., 2014). The σ^54^, which controls stress related genes including those involved in nitrogen fixation, nutrient starvation, infection, and other cellular stresses, forms a class of its own (Buck et al., 2000, Merrick, 1993). σ^54^ recruits RNAP to its promoter sites via binding to the −12 and −24 consensus promoter sequences and forms a stable RPc that rarely spontaneously isomerizes to RPo. Instead, it requires ATPase activators bound remotely upstream of RPc to convert to RPo (Buck et al., 2000). The σ^54^ system thus resembles eukaryotic RNA Pol II systems where recruitment to the promoter to form RPc and isomerization to RPo are decoupled and the isomerization requires ATP-dependent structural transitions (Nogales et al., 2017, Schilbach et al., 2017). Forms of RPc have been captured for the human and yeast RNAP Polymerase II (He et al., 2016, Murakami et al., 2015, Plaschka et al., 2016). Some forms of RPo have also been captured for human and yeast RNAPII as well as bacterial RNAP in complex with σ^70^ (Bae et al., 2015, Davis et al., 2015, He et al., 2016, Hubin et al., 2017, Plaschka et al., 2016, Zhang et al., 2012, Zuo and Steitz, 2015). Using the unique properties of the σ^54^ system, we have recently determined the structures of a bacterial RPc and one transcription intermediate complex (RPi), using the activator phage shock protein F (PspF) in complex with an ATP hydrolysis transition state analog, ADP.AlFx (Glyde et al., 2017). A number of initial transcribing complexes (RPitcs) have been reported which provided insights into RNA synthesis when RNAP is promoter bound (Bae et al., 2015, Basu et al., 2014, Cheung et al., 2011, Zhang et al., 2012, Zuo and Steitz, 2015). However, apart from a low-resolution (5.5–6 Å) crystal structure of *E. coli* RNAP-σ^70^(Zuo and Steitz, 2015) and a structure obtained from *in crystal de novo* synthesis reaction (Basu et al., 2014), synthetic RNA sequences were base paired with a section of pre-opened transcription bubble to form RPitcs for structural studies. Consequently, we have limited information on the organization of DNA and RNAP during initial *de novo* RNA synthesis, now addressed in this study.

**Figure 1:**
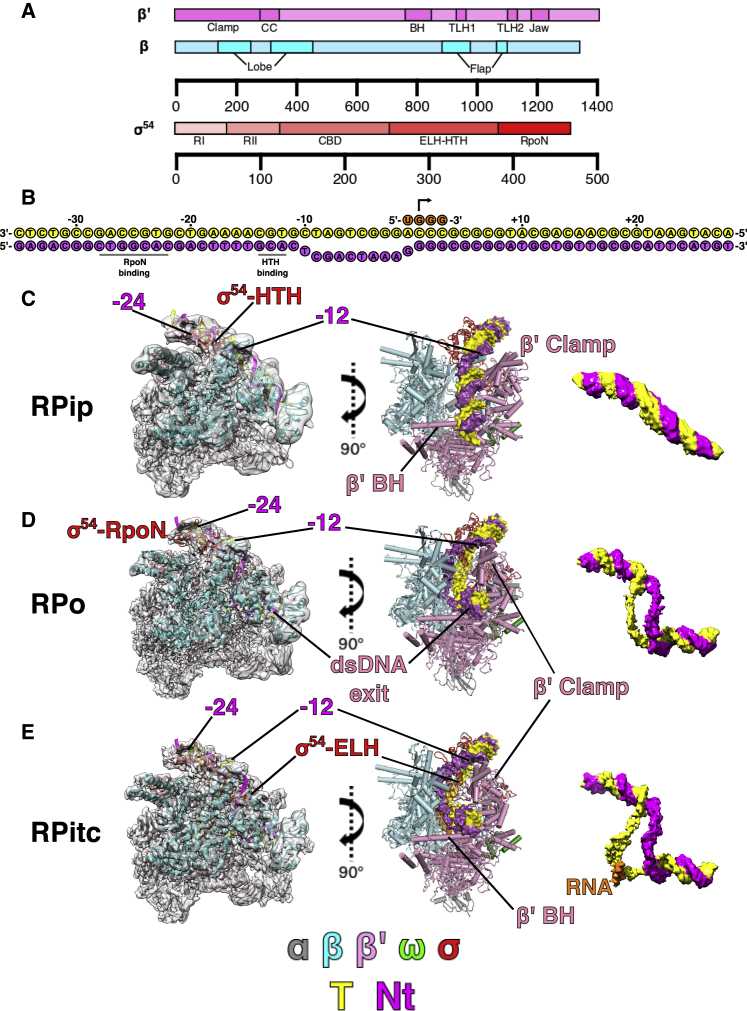
Cryo-EM Reconstructions and Structural Models of RPip, RPo, and RPitc (A) Schematics of RNAP β, β′, and σ^54^ used in the study. (B) Schematics of the DNA and RNA sequences used in this study. σ^54^ binding sites are labeled. (C–E) 3D reconstructions and structural models in two orthogonal views. Also shown is the corresponding nucleic acid density. (C) RPip; (D) RPo; (E) RPitc. See also Figures S1–S3.

Our previous work on RPc and RPi identifies important functional domains in σ^54^ and explains how σ^54^ inhibits transcription and the roles of activator in relieving the inhibition (Glyde et al., 2017, Yang et al., 2015). We showed that σ^54^ region I (RI, ∼residues 15–30) and the extra-long helix-helix turn helix (ELH-HTH, ∼residues 300–390) (Figure 1A) interact and form a blockage to prevent DNA from entering the RNAP cleft. Interactions with the activator protein partially remove this blockage (Glyde et al., 2017, Yang et al., 2015). The requirement of the activator can be alleviated using a σ^54^ mutant (R336A), provided the transcription bubble is pre-opened (Chaney and Buck, 1999). In order to understand how transcription is initiated and how initial *de novo* synthesis is carried out, we prepared samples to capture the RPo and the RPitc state by binding σ^54^ R336A to a promoter (−35 to +28) with a pre-opened transcription bubble (mismatch between −10 and −1 by mutating the non-template [NT] strand) to form RPo and then adding UpG di-nucleotide as well as GTP, to allow *de novo* synthesis of a −1 UpGGG +3 RNA to form RPitc (Figure 1A). In so doing, we also capture a new intermediate complex where DNA is caught at the entrance of the RNAP cleft (Figures 1 and S1–S3). This structure resembles the previously proposed intermediate state (RPi1) based on DNA footprinting data on σ^70^-dependent transcription (Craig et al., 1998, Schickor et al., 1990, Sclavi et al., 2005). Importantly, these structures support a coupled DNA load and unwind model and provide detailed structures and mechanism during transcription initiation that could be applicable to all multisubunit RNAPs.

## Results and Discussion

### Structure of RNAP with Partially Loaded DNA

**Figure 2:**
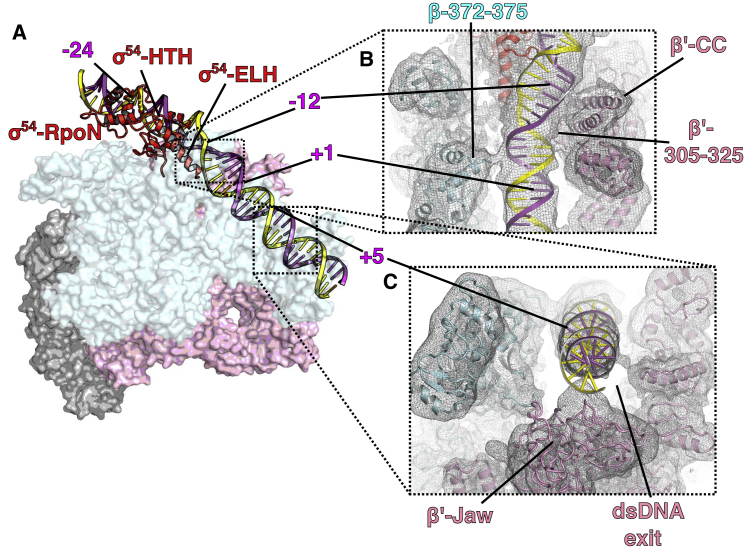
DNA Stabilization in RPip (A) Overall path of DNA with –24 and –12 interacting with RpoN and HTH of σ^54^. (B) DNA (–10 to +1 region) is stabilized by β′, β, and σ^54^ ELH. (C) Downstream DNA sits above β′ jaw domain. See also Figure S4.

In both RPo and where nucleotides were added to RPo, in addition to RPo or RPitc, another conformation in which DNA is yet to fully enter into the RNAP cleft was also captured at 4.1 Å resolution (Figure 1C). In this structure, DNA is caught at the RNAP cleft and likely represents an intermediate state *en route* to RPo (see discussion below), and we term it RPip (for partially loaded DNA). The resolution for DNA is poorer compared to RNAP (Figure S2B), suggesting that there is some conformational flexibility in the DNA in this state. As expected, σ^54^ C-terminal RpoN domain interacts with −24 promoter region and ELH-HTH interacts with −12 region (Figure 2A). Interestingly, we observe an ∼30° bend/kink in DNA just downstream of −12/−11 toward the RNAP cleft, and the density is sufficient to accommodate both DNA strands, suggesting that the two strands are still in close proximity even though they are not base-paired between −10 and −1 (Figure 1B). There are extensive interactions between RNAP-σ^54^ and DNA that facilitates the −10 bend/kink. The regions between −10 and −1 are in fact sandwiched between σ^54^-ELH and RNAP β-lobe on one side and β′ coiled coil within the clamp on the other side (Figure 2B). In particular, the highly positive charged β′ coiled coil and loop (residues 305–325) within the clamp and the proline/glycine loop (^372^PGEP^375^) in the β-lobe are positioned to stabilize the DNA phosphor-backbone between −10 and −1, suggesting that they play an important role in stabilizing RPip and potentially guiding the DNA to enter the RNAP cleft (Figure 2B). The kink signifies the point at which the DNA is turning for entering the RNAP cleft and the transcription bubble formation starts. To accommodate the DNA path, σ^54^ ELH, which is single long α helix between residues 317–355 in RPc and RPi, appears to be shorter (no clear density for residues 317–330 in the helical conformation), suggesting that ELH undergoes conformational changes during DNA loading. Indeed, there is additional density that suggests a different trajectory for residues 317–330 of the ELH (Figure S4A), although the quality of the density prevented a structural model to be built. DNA downstream of +1 only interacts with RNAP at the β′ jaw domain, which forms a basin to accommodate downstream DNA (Figure 2C). The lack of specific interactions between DNA and protein might contribute to the conformational flexibility and thus reduced resolution.

### Structure of the Open Promoter Complex RPo

**Figure 3:**
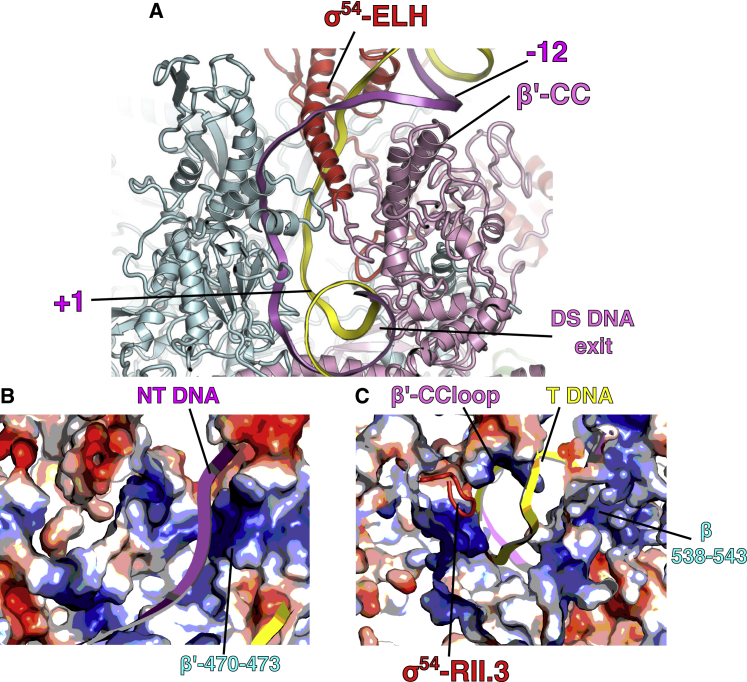
DNA Interactions in RPo (A) DNA enters the RNAP cleft and the T and NT strands are separated by ELH. (B) NT strand is stabilized by positively charged residues. (C) T strand in a tunnel formed by β, β′, and σ^54^ RII.3. See also Figure S4.

Our cryoelectron microscopy (cryo-EM) reconstruction of RPo at 3.4 Å (Figures 1D and S1A; Table 1) shows that most of the RNAP has clear density for side chains (Figure S3). DNA is visible with a clear trace for the T and NT strands (Figure 1C). The DNA strands are first separated from around −11 with the NT strand draping over the σ^54^ ELH toward the β subunit (Figure 3A). Interestingly, the ELH appears longer compared to that in RPip with density for residues up to 324 although is still short of the fully extended ELH. The transcription bubble contains 13 nt (−11 to +2) of single-stranded DNA (ssDNA) with dsDNA downstream of +3 (Figure 3A). The NT strand follows a path that is lined with positively charged residues (Figure 3B), whereas the T strand goes through a tunnel formed by β (residues 538–543), β′ coiled-coil loop (residues 318–323) and σ^54^ RII.3 (residues 107–112) (Figure 3C). The downstream ss-dsDNA junction is very similar to those observed in σ^70^ RPo with +2 NT base inserted into a hydrophobic pocket formed by β subunit and +1 NT base forms hydrophobic-base interactions with W183 of β subunit (Figure S4B) (Zhang et al., 2012). It is noteworthy that, despite the structural and functional differences between σ^54^ and σ^70^, the transcription bubble architecture remains the same, opening at −11 at the upstream edge while returning to dsDNA at +3. However the DNA path as well as the ways the transcription bubble are stabilized at the upstream edge are different (Figure S4C), with σ^70^ using conserved Trp residues to stabilize the flipped out −11 NT base, while σ^54^ uses its ELH to physically separate the two strands (Figure 3A) (Bae et al., 2015, Feklistov and Darst, 2011, Zhang et al., 2012). Comparisons with σ^70^ promoter complex now further explain the different promoter recognitions between σ^70^ and σ^54^. The σ^54^ HTH and RpoN box interact with −12 and −24 regions and σ^70^ regions 3 and 4 recognize −10 and −35 promoter regions, respectively. σ^54^ HTH and σ^70^ region 3 are similarly located, toward the β side of the cleft, thus bringing −12 (σ^54^) and −10 (σ^70^) promoter elements to similar locations relative to the RNAP cleft (Figures S4D and S4E). However, the σ^54^ core binding domain (CBD) occupies similar location as σ^70^ region 4, on the β′ side of the cleft. Instead the RpoN box is located on the β-side, allowing it to interact with −24 (instead of −35) regions (Figures S4D and S4E).

**Table 1:** Cryo-EM Data Collection, Processing, and Refinement Statistics

	RPo	RPip	RPitc
**Data Collection**			

Total particles	257,434	639,379	639,379
Pixel size (Å)	1.06	1.08	1.08
Defocus range (μm)	−1.2 to −3.2	−1.2 to −3.2	−1.2 to −3.2
Voltage (kV)	300	300	300
Electron dose (e^−^ Å^−2^)	45	48	48

**Reconstruction (RELION)**			

Particles	79,678	53,709	89,996
Resolution (Å)	3.4	4.1	3.7

**Refinement**			

Resolution	3.4	4.1	3.7

**Root-Mean-Square Deviation**			

Bond length (Å)	0.003	0.002	0.002
Bond angle (°)	0.678	0.638	0.622

**Ramachandran Plot**			

Favored regions (%)	88.46	89.21	89.45
Allowed regions (%)	10.66	10.08	9.88
Outlier	0.89	0.71	0.67

**Validation**			

All-atom clashscore	12.12	11.56	10.12
Rotamer outliers (%)	0.08	0.12	0.04
C-beta deviations	0	0	0

### Structure of the *De Novo* Transcribing Complex

**Figure 4:**
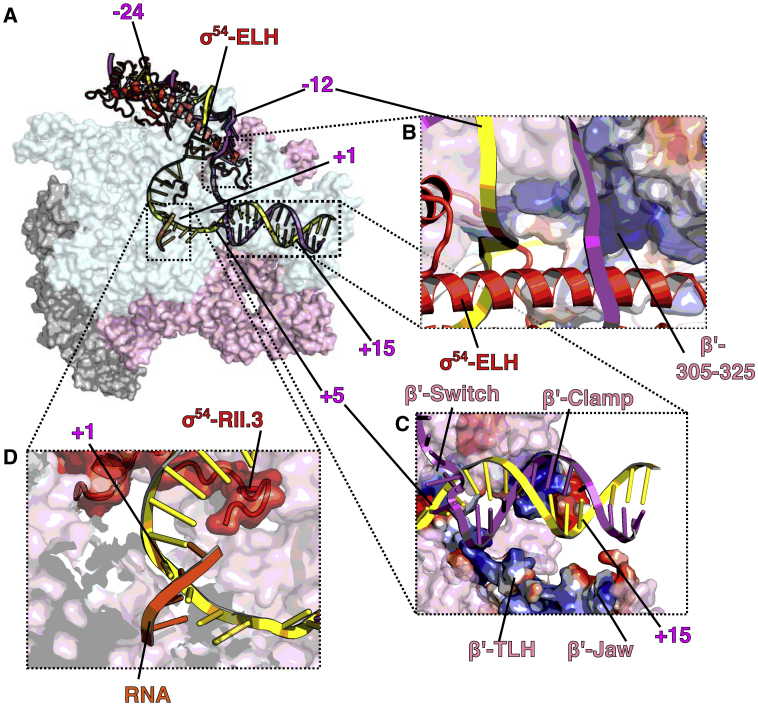
DNA and RNA in RPitc (A) Overall DNA and RNA path. (B) Upstream bubble, strands separated by ELH with T further stabilized by β′ coiled-coil loop. (C) Downstream DNA stabilized by β′ clamp and β′ jaw. (D) Synthesized RNA and the location of σ^54^ RII.3. See also Figures S5 and S6.

Where nucleotides were added to RPo, we obtained a structure at 3.7 Å of the initial *de novo* synthesizing complex (RPitc), which has density for the whole transcription bubble, the newly synthesized RNA, as well as dsDNA both upstream and downstream of the transcription bubble (Figures 1E and 4A). In the RPitc, the upstream ss-dsDNA junction is similar to RPo but now with clearer density for T strand (Figure 4B). The β′ coiled-coil loop is now positioned in-between the T strand, RNA and σ^54^ RII (Figure S5A). The downstream dsDNA is stabilized by residues from β′ switch 1 region (1325–1328), β′ clamp (residues 207–212), β′ trigger loop/helix (1145–1150), and β′ jaw domain (residues 1170–1175) (Figure 4C). In the RPitc, the transcription bubble has extended to 15 nt, in agreement with two additional nucleotides being added to the initiating −1+1 UpG di-nucleotide. T strand now is pushed further back into the channel and is stabilized by β R1269, K1262, and β′ R346 (Figure S5B). The T strand is in an expanded conformation compared to RPo (Figure S5C). There is a cavity behind the T strand, 6–8 nt upstream of the active site, as well as a cavity just above the bridge helix, that could accommodate scrunching of T and NT strands, respectively, upon further nucleotide synthesis (Figures S5D and S5E), as proposed based on single-molecule experiments (Kapanidis et al., 2006, Revyakin et al., 2006). Interestingly, we observe density corresponding to σ^54^ RII.3 close to T strand and the newly synthesized RNA (Figure 4D). RII.3 contains acidic patches that are connected to σ^54^ CBD via the RNA exit channel (Yang et al., 2015). The structure here is consistent with a role for RII.3 in stabilizing and guiding RNA toward its exit channel (Figure 4D). Indeed, σ^70^ region 3.2 occupies the same location and is proposed to play similar roles (Bae et al., 2015, Zuo and Steitz, 2015).

We see clear density for the 4 nt RNA that base pair to the T strand (Figures 1E, 4D, and S3E). To verify that under the sample conditions for cryo-EM, the system is competent for RNA synthesis and to confirm the precise RNA being synthesized, we used small primed RNA (spRNA) assays and RT-PCR followed by cloning and sequencing. Our data confirm that, under these conditions, a major species of four nucleotides with a sequence of UGGG is synthesized (Figures S6A–S6C). Our structure reveals that the RNA is in a post-translocation position, and the trigger loop is accordingly in an open conformation with a shorter trigger loop helix **(**Figure S6D) (Vassylyev et al., 2007, Wang et al., 2006). Interestingly, in the RNAP-σ^70^ *de novo* synthesizing RPitc crystal structure, RNA lies in a pre-translocation position, with the trigger loop is in a closed conformation (Figure S6E) (Zuo and Steitz, 2015). The differences in trigger loop conformations are further amplified in the different locations of β′ trigger loop insertion and jaw domain (Figure S6F), which interacts with downstream dsDNA. The reasons for these structural differences are unknown but could be due to different constraints imposed in crystallization or cryo-EM. However, the structural changes and domain movements observed between these structures could well represent coordinated changes required for RNA/DNA translocations during RNA synthesis (Ederth et al., 2002, Wigneshweraraj et al., 2006).

### Comparisons of RPip and RPo Suggest a Coupled DNA Load and Unwind Model

Comparisons of structures of the RPip and RPo reveal large conformational changes in RNAP that could accommodate DNA loading as well as changes in σ^54^ that could facilitate the loading and transcription bubble stabilization. Footprinting and kinetic studies with the σ^70^ holoenzyme have suggested the existence of two intermediate states prior to RPo: RPi1 and RPi2 (Craig et al., 1998). RPi1 is proposed to be a state where DNA adopts a sharp (∼90°) bent around −12/−11 and places the downstream DNA (−5 to +20) loosely in the β and β′ jaws, although this entire region in some promoters is not protected by DNase I footprinting (Craig et al., 1998, Saecker et al., 2002). Time-resolved hydroxyl footprinting on σ^70^-dependent system confirmed the existence of intermediate states with similar properties with DNA between −50 to −9 and/or +2 of being protected (Schickor et al., 1990, Sclavi et al., 2005). Although the RPip state reported here has not been established definitely as a bona fide on-pathway intermediate, RPip is broadly consistent with the proposed RPi1. Indeed, in RPip, we observe a DNA bending (∼30°) around −12/−11 and the DNA (−10 to −5, Figure 2B) was sandwiched between β′ clamp on one side and σ^54^ ELH and β lobe on the other side although downstream DNA (to ∼+15) sits loosely above the jaw domains. The difference in DNA bending between what we observe in RPip and those measured in σ^70^ DNA footprinting studies could reflect the different upstream DNA paths between σ^54^ and σ^70^ systems, with >30° tilt between σ^70^ and σ^54^ (Figures S4D and S4E). RPi2 is proposed to be a short-lived state in which DNA has been loaded into the cleft before converting to a stable RPo (Craig et al., 1998, Saecker et al., 2002). Conversion from RPi1 to RPi2/RPo is proposed to be a rate-limiting step and involves large conformational changes in RNAP (Craig et al., 1998), and the isomerization step in σ^54^-dependent system is reported to be slow (Friedman and Gelles, 2012). The large conformational changes we observe between RPip and RPo could thus represent those changes proposed between RPi1 and RPi2/RPo.

**Figure 5:**
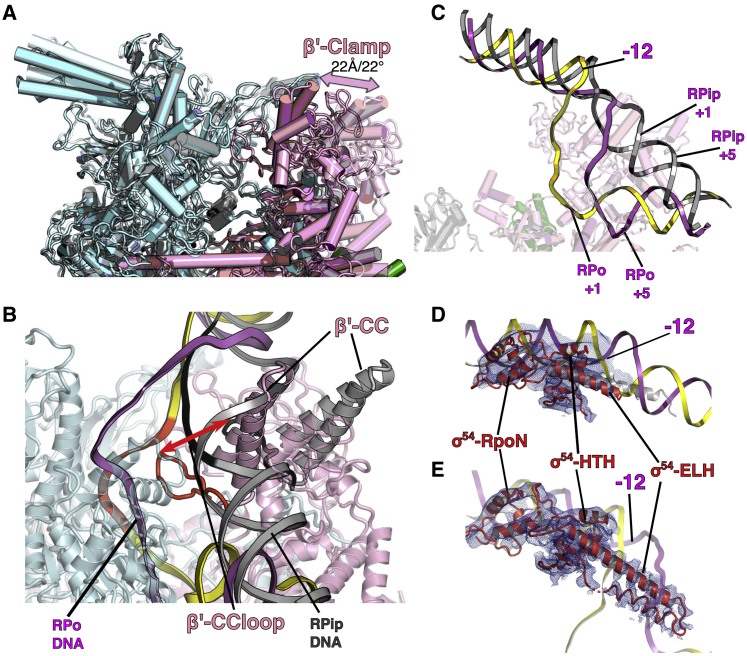
Comparison of RPo (Colored) and RPip (Pale Colored) and the Mechanism of DNA Loading Structures are superposed on their bridge helix (BH). (A and B) Clamp movement (A) and β′ coiled-coil rotation (B) results in a downward movement of β′ coiled-coil loop (305–325) that interacts with DNA T strand in both RPip and RPo. The loop and interacting DNA are colored black for RPip and red for RPo. (C) Conformational changes in DNA. (D and E) σ^54^ RII.3 ELH in RPip (D) and RPo (E). Density displayed as mesh. See also Figure S7.

The RNAP clamp is wide open in RPip while closed in RPo (Figure 5A; Videos S1 and S2). The clamp movement pivots around the base of the cleft (switch region) with ∼22° rotation, with largest movements (>20 Å) at the tip of the pincer (Figures 5A and 5B). Clamp closure would thus lead to a downward push, favoring DNA to be moved in. Given the constraints imposed on the DNA, by σ^54^ in the upstream and by β′ jaw in the downstream, the movement of the clamp and DNA would result in a significant kink and unwinding in DNA, thus driving the strand separation for a transcription bubble to form. Indeed, β′ coiled-coil loop (residues 305–325) within the clamp interacts with DNA (−7/−6 T strand) in RPip (Figure 2B). These interactions are maintained in RPo and RPitc. The rotation of β′ coiled coil and subsequent downward movement of the coiled-coil loop into the RNAP active channel thus help the delivery of the DNA into the cleft and stabilization of the two DNA strands (Figure 5B). Comparisons of the DNA positioning in RPip and RPo indeed confirm that upstream (−12) and downstream nucleotides are similarly located relative to RNAP while the DNA path in RPo suggests the need for unwinding and separation of dsDNA (Figure 5C). Clamp closure thus appears to be an obligatory step in DNA loading as well as promoter melting.

**Figure 6:**
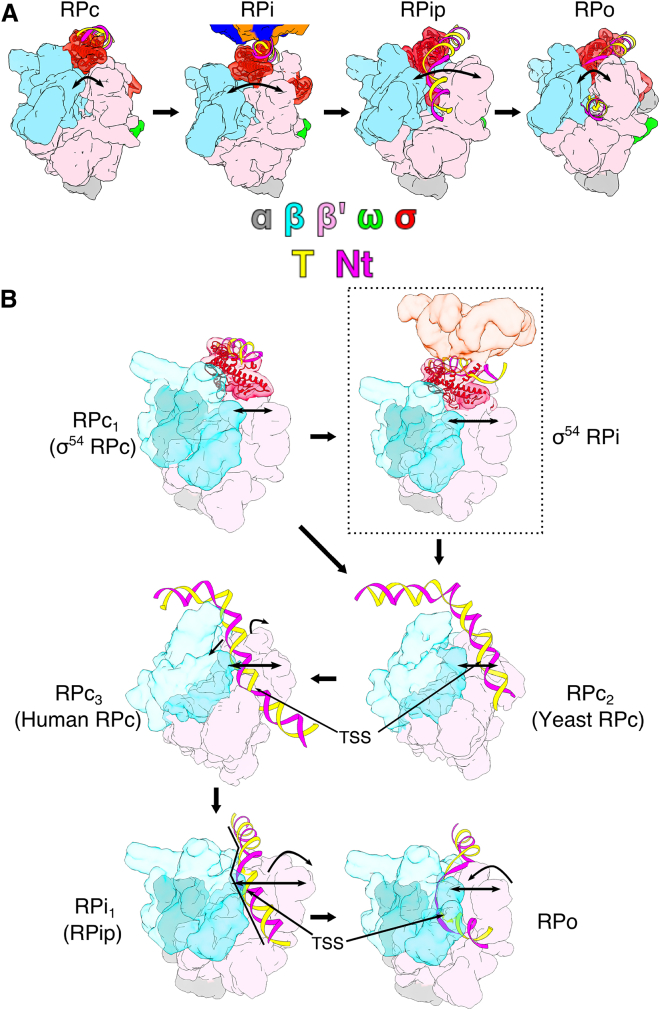
Conformational Changes during Isomerization and a Proposed “Coupled Load and Unwind” Initiation Model (A) Conformational changes from RPc to RPo in σ^54^ system. RNAP cleft opens initially from RPc to RPi to RPip before closes down in RPo. These are correlated with DNA path and σ^54^ conformations. RNAP in surface representation, σ^54^ and DNA in ribbon cartoon. Arrows indicate clamp opening. (B) Proposed transcription initiation model. The initial closed complex is system specific; shown here is σ^54^ RPc (PDB: 5NSR, labeled RPc1) where DNA is high above the RNAP cleft. RNAP is then converted to RPi in σ^54^ system but could go to a conformation similar to those captured in yeast Pol II closed complex (RPc2, PDB: 5FZ5), before transition to RPc2 as captured in human Pol II closed complex (PDB: 5IYA) where the RNAP clamp is slightly open and DNA starts to bend into the RNAP cleft. In RPi1 (RPip), DNA makes a 30° kink in σ^54^ (>60° in σ^70^) and is at the entrance to the RNAP cleft with the clamp wide open. Clamp closure to RPo causes the DNA to load and unwind. See also Figure S7.

Video S1. Morph Video Showing Conformational Changes during Isomerization from RPc to RPi, RPi to RPip, RPip to RPo, and RPo to RPitc to Illustrate the Dynamics of DNA, Sigma, and DNA during DNA Loading; Related to Figures 5 and 6 Color scheme same as in Figure 1. β, cyan; β′, salmon pink; α, light gray; σ^54^, red; DNA T strand, yellow; NT strand, magenta. Activators in RPi were removed and DNA was extended using B-DNA to the same length for all the structures. Sections of the NT strand were removed during the transition from RPip to RPo due to the uncertainty in the exact path of the DNA, which is likely to be non-linear.

Video S2. Same as Video S1, Viewed from the β Subunit Side; Related to Figures 5 and 6

Apart from significant conformational changes in RNAP, changes are observed in σ^54^ between RPip and RPo. In RPip, we observe clear density for the C-terminal part of ELH, which contains HTH bound to −12/−11 promoter DNA, while no clear density that could accommodate a fully extended ELH helix in the N-terminal part, which would indeed cause steric clash with DNA (−6 regions) (Figure 5D). This strongly suggests that N-terminal part of ELH undergoes conformational changes during DNA loading. Indeed, there is density that suggests an alternative path trajectory for this part of the σ^54^ ELH structure (Figure S4A). Importantly, upon DNA loading and transcription bubble stabilization, as observed in RPo and RPitc, the ELH returns to an extended helix acting as a saddle to separate the two DNA strands (Figure 5E). The dynamic ELH thus plays a crucial role in coordinating DNA loading and transcription bubble stabilization.

The stabilization by ELH in the σ^54^ system is in stark contrast to that of σ^70^ system, which utilizes Trp residues to intercalate into a small melted out piece of promoter DNA. The differences offer insights into the different requirements for isomerization in the two systems, with the strict requirement of activator proteins in σ^54^-dependent promoters. The transcription bubble stabilization by ELH requires an extensively melted DNA region, which is propagated by activator interactions with RI in σ^54^-dependent promoters (Glyde et al., 2017). Activators thus play two vital roles: relieving the inhibition imposed by RI and ELH and weakening the double-stranded DNA downstream of −12. In σ^70^, the observed conformational changes between RPip and RPo in RNAP are sufficient to drive DNA delivery and separation that are then stabilized by Trp intercalation with the promoter DNA.

Our data lead to the proposal that DNA loading and promoter melting are integrated processes (“coupled DNA load and unwind” model) and are consistent with the proposed intermediate states from earlier footprinting and kinetic studies (Craig et al., 1998, Sclavi et al., 2005). RPip could represent RPi1, whereas RPi2 could be the state when DNA has just been loaded into the cleft and strands are separated, but before single-stranded DNAs are stabilized by interactions with RNAP as observed in RPo.

Our “coupled DNA load and unwind” model contrasts with other models that suggest melting proceeds loading (“unwind first, then load” model) (Feklistov et al., 2017). The “unwind first, then load” model was based primarily on three arguments. (1) The RNAP cleft in RPo is too narrow to accommodate a dsDNA. The authors thus proposed that dsDNA must be melted out first and the DNA single strands were subsequently pulled into the channel by the highly positively charged residues. Our structure of RPip demonstrates that the cleft opens up before it closes down during DNA loading, thus permitting the dsDNA to enter the cleft where cleft closure subsequently induces DNA melting. (2) A steep temperature dependence of the promoter melting step. This observation is also consistent with our model where the process of cleft closure/dynamics and DNA opening as well as σ^54^ ELH conformational dynamics, which are coordinated actions to enable DNA loading and melting, will all be temperature dependent. Indeed, large conformational changes have been observed between RPi1 and RPi2 (Saecker et al., 2002) in which RPi2 contains a full opened bubble (Gries et al., 2010). These observations are inconsistent with the “unwind first, then load” model in which there were modest conformational changes between RPi1 and RPi2 (Feklistov et al., 2017). (3) The observation that melting does not proceed downstream of −10 element if the β lobe domain is deleted. We find that the β lobe indeed plays an important role in RPip stabilization (Figure 2B). The data are thus also consistent with our model, that the transition from an open clamp in RPip (RPi1) to a closed clamp in RPi2/RPo drives the loading and melting.

In addition, our “coupled load and unwind model,” where loading process induces the opening of the transcription bubble, supports and is consistent with earlier kinetics data, which showed that RNAP opens the entire transcription bubble (−11 to +2) in a single step (Gries et al., 2010). Furthermore, footprinting data showed that binding of promoter DNA in the cleft (in RPi1 or RPip) triggers large-scale conformational changes, which likely induces cleft closure that leads to RPi2, and the conformational changes occur before the DNA melting (Craig et al., 1998). These earlier footprinting data are fully consistent with our model and, however, are inconsistent with many aspects of an “unwind first, then load” model (Feklistov et al., 2017).

### Mechanism of Transcription Initiation

Our new structures and previous RPc and RPi structures now allow us to describe the conformational pathway during the isomerization from RPc to RPo. DNA distortions downstream of −12 are initiated in RPc, via interactions with the N-terminal RI and HTH of σ^54^ (Glyde et al., 2017). These distortions are important for the engagement with AAA+ activators, which in turn promote and propagate strand separation (Glyde et al., 2017). The RPip structure here suggests that during loading, the DNA enters the RNAP cleft via a bend/kink around −10, and this is coordinated with RNAP clamp opening and σ^54^ ELH conformational changes. In RPip, DNA is caught between β and β′ clamps and ELH does not extend to a full helix, which would interfere with DNA. Upon clamp closure, the lowering of the DNA into the cleft will be accompanied by strand separation that is then stabilized by ELH, now in an extended conformation and acts as a saddle for the two DNA strands to drape around.

The RNAP clamp is initially closed in RPc, it opens up in RPi and RPip, with the largest opening captured in RPip (Figure 6; Videos S1 and S2), presumably to facilitate initial DNA loading. Upon loading deep into the RNAP channel as observed in RPo and RPitc, the RNAP clamp closes down, returning to a conformation similar to that of RPc (Figure 6; Videos S1 and S2). Our structures thus reveal that at least two distinct transcriptional intermediate states (RPi and RPip) exist in σ^54^ system and they both have an open clamp and are significantly different from those of RPc and RPo. On the other hand, RNAP structures in RPc and RPo are remarkably similar, highlighting the dynamic nature of the isomerization process and the importance in capturing intermediate states in understanding the transcription initiation process. The range of changes are consistent with those observed in single-molecule fluorescence resonance energy transfer (FRET) experiments (Chakraborty et al., 2012), with RPip showing a clamp opening of more than 20° compared to those of RPc or RPo, resulting in >20 Å widening of the cleft at the widest point (Figure 5A).

In the σ^54^ system, changes to the DNA paths are directed by σ^54^ ELH, which in turn is affected by clamp movement. The ELH connects the two pincers in RPc and RPi, with its N terminus interacting with β′ coiled-coil domain within the β′ pincer while its C terminus interacting with β-flap of the β pincer (Figures S7A and S7B). Indeed, the ELH acts as a crowbar and moves with the clamp (Figures S7A and S7B). In RPip, the β′ clamp opens widely, losing interactions with ELH (Figure S7C). ELH now swings into the wide-open cleft, bringing DNA with it. The changes in ELH from RPi to RPip involve an ∼60° rotation into the cleft and an altered N-terminal structure. The rotation in ELH results in an ∼30° bend/kink in DNA, directing DNA toward the cleft, although this is not fully entered yet (Figures 6B, S7B, and S7C). The DNA path is further guided and stabilized by positive charges in β′ clamp and β′ jaw (Figures 3B and 3C). Upon clamp closure (RPip to RPo, Figures 6B, S7C, and S7D), DNA fully enters the cleft, accompanied by strand separation and the transcription bubble is now irreversibly separated by ELH. Upon *de novo* RNA synthesis, T-strand nucleotides are moved toward the back of the RNAP channel and stabilized by positively charged residues and the newly synthesized RNA is then guided by σ^54^ RII.3 toward the exit channel.

Our structural studies here now provide direct evidence that transcription initiation is indeed a multi-step and highly dynamic process. Coordinated conformational changes and movements of the RNAP clamp, σ, and promoter DNA are observed along the pathway leading to *de novo* RNA synthesis. Clamp opening and closing have been observed in FRET experiments (Chakraborty et al., 2012), but now we provide detailed structural snapshots that correlate the clamp opening and closing with the isomerization process. DNA loading requires a dynamic RNAP clamp that opens up to allow the initial loading and then closes down to complete the loading. Transcription bubble dynamics as well as σ^54^ ELH conformational dynamics orchestrate the loading and bubble capture. Although it has not been firmly established that the details of the isomerization from RPc to RPo are conserved between σ^54^- and σ^70^-dependent transcription, and the lack of structural information on σ^70^ closed and intermediate complexes, the resemblance between RPip and RPi1 and the proposed “coupled DNA load and unwind” model from this work and those based on footprinting and kinetic data on the σ^70^ system suggest shared requirements for large-scale dynamic changes in RNAP, σ, and DNA.

Interestingly, closed complexes of human and yeast RNAP II (Pol II) have displayed slightly different conformations in terms of DNA paths and the clamp openings and these two closed complex structures have been suggested to represent slightly different conformations along the isomerization pathway (He et al., 2016, Plaschka et al., 2016). The clamp in the yeast Pol II closed complex is in a closed conformation, similar to those observed in our RPc and closed complex in yeast Pol III (Glyde et al., 2017, Vorländer et al., 2018). In human Pol II closed complex, the clamp is slightly more open and DNA bends around −10 toward the cleft. In our RPip structure, the clamp is opened up further (>20° rotation and >20 Å displacement compared to RPc), resulting in a significantly wider cleft to accommodate the DNA at the point of being loaded into the cleft and is consistent with the wide-open conformation observed in single-molecule FRET experiments (Figure S7) (Chakraborty et al., 2012). These structures lead to the proposal that the conformations of yeast Pol II closed complex (and in other closed complexes), human Pol II closed complex, and RPip represent conformations from RPc to RPi1 in all the multisubunit RNAPs. The RPi captured for the σ^54^ system (Figure 6) could be particular for σ^54^-dependent transcription due to the unique binding mode of σ^54^ to the promoter DNA and its requirement of activators. All the open-complex structures reported so far, from bacteria to human, have similar cleft conformations with a closed clamp (Abascal-Palacios et al., 2018, Bae et al., 2015, He et al., 2016, Plaschka et al., 2016; Vorlander et al., 2018; Zhang et al., 2012, Zuo and Steitz, 2015). Our data here, supported by kinetic data on σ^70^ system and the available structures of yeast and human Pol II closed complex and open-complex structures in both bacteria and eukaryotic Pol II and Pol III systems, support a general model for transcription initiation that involves clamp opening coupled with DNA bending at −10 to enable DNA being lowered into the RNAP cleft. Subsequent clamp closure causes DNA to be pushed into the cleft while being unwound into a transcription bubble (coupled DNA load and unwind model, Figure 6). However, how such system-specific changes in structure are coordinated and regulated require further studies of the regulation of a range of system-specific transcription intermediate complexes.

## STAR★Methods

### Key Resources Table

**Table:** 

REAGENT or RESOURCE	SOURCE	IDENTIFIER
**Bacterial and Virus Strains**		

*E. coli* BL21 (DE3)	NEB	C2527H

**Chemicals, Peptides, and Recombinant Proteins**		

Plasmid pGEMABC (encoding full length of *E. coli* rpoA, rpoB and rpoC)	Yang et al., 2015	addgene #45398
Plasmid pACYCDuet-omega (encoding full length rpoZ of *E. coli*)	Yang et al., 2015	N/A
Plasmid pET28b-σ54_R336A_ (N-terminal 6 × His tag, encoding full length of σ54_R336A_ from K. pneumoniae M5A1)		N/A

**Deposited Data**		

Intermediate Partially loaded DNA complex (RPip)	This work	EMD-0002, PDB 6GH6
Open promoter complex (RPo)	This work	EMD-0001, PDB 6GH5
Initially Transcribing complex (RPitc)	This work	EMD-4397, PDB 6GFW

**Oligonucleotides**		

63 nucleotide *nifH* promoter template strand 5′-ACATGAA TGCGCAACAGCATGCGCGCCCAGGGCTGATCGTGCAAAA GTCGTGCCAGCCGTCTC-3′	This work	
63 nucleotide *nifH* promoter non-template strand with −10/-1 mismatch 5′-GAGACGGCTGGCACGACTTTTGC ACTCGACTAAAGGGGCGCGCATGCTGTTGCGCATTCATGT-3′	This work	

**Software and Algorithms**		

COOT	Emsley et al., 2010	https://www2.mrc-lmb.cam.ac.uk/personal/pemsley/coot/
Relion	Scheres, 2012	http://www2.mrc-lmb.cam.ac.uk/relion/index.php?title=Main_Page
	Scheres, 2012	
Phenix real_space_refine	Afonine et al., 2012	https://www.phenix-online.org/documentation/reference/real_space_refine.html
Refmac	Murshudov et al., 1997	
Motioncor2	Zheng et al., 2017	http://msg.ucsf.edu/em/software/motioncor2.html
Gctf	Zhang K, 2016	https://www.mrc-lmb.cam.ac.uk/kzhang/Gctf/
Gautomatch	N/A	https://www.mrc-lmb.cam.ac.uk/kzhang/
Cryosparc	Punjani et al., 2017	https://cryosparc.com/
MDFF	Trabuco et al., 2009	http://www.ks.uiuc.edu/Research/mdff/

### Contact for Reagent and Resource Sharing

As Lead Contact, Xiaodong Zhang is responsible for all reagent and resource requests. Please contact Xiaodong Zhang at xiaodong.zhang@imperial.ac.uk with requests and inquiries.

### Method Details

#### Sample preparation

*E. coli* RNA polymerase (RNAP) and *K.* pneumoniae σ^54^_R336A_ were expressed and purified as described previously (Yang et al., 2015). The holoenzyme was formed by incubating RNAP with a four-fold molar excess of σ^54^ prior to size exclusion chromatography (Superose 6 10/300 – GE Healthcare). The holoenzyme was incubated with 1.4 times molar excess of DNA (−35 to +28 with a mismatch between −10 and −1 on the non-template strand) with the following sequence:

Template strand
5′ ACATGAATGCGCAACAGCATGCGCGCCCAGGGCTGATCGTGCAAAAGTCGTGCCAGCCGTCTC-3′,
Non-template strand
5′-GAGACGGCTGGCACGACTTTTGCACTCGACTAAAGGGGCGCGCATGCTGTTGCGCATTCATGT-3′

In the case of the RPitc, 1 mM UpG dinucleotide and 1 mM GTP were also added. Incubation was for 1 hour at 4**°**C. Samples were then buffer exchanged using Zeba Spin Desalting Columns into the final buffer used for cryo-EM (10 mM Tris-HCl pH 8.0, 150 mM NaCl, 10 mM MgCl_2_).

#### Electron microscopy

3 μL of samples at a concentration of 0.5 mg/ml were applied to R2/2 holey carbon grids (Quantifoil). Vitrification was carried out using a Vitrobot Mark IV (FEI) at 4°C and 95% humidity for 0.5 s with a blotting force of −5.

All data were collected on a Titan Krios using EPU (FEI) operated at 300 KeV and a K2 Summit direct electron detector (Gatan) using a defocus range of −1.2 μm to −3.2 μm. The RPo dataset was collected at eBIC (Diamond Light Source, UK) with a pixel size of 1.06 Å/pixel and a total dose of 45 e^−^/Å^2^ fractionated into 25 frames (1.8 e^-^/Å^2^/frame). The RPitc dataset was collected at the Francis Crick Institute with a pixel size of 1.08 Å/pixel and a dose of 48 e^-^/Å^2^ over 30 frames (1.6 e^-^/Å^2^/frame). A total of 916 micrographs were collected for the RPo and 4205 for the RPitc.

#### Image processing

Similar image processing procedures were used for both datasets and are summarized in Figures S1 (RPo) and S2 (RPip and RPitc). Frame alignment and dose weighting were carried out by MotionCor2 (Zheng et al., 2017) before estimating CTF parameters using Gctf (Zhang, 2016) and particle picking with Gautomatch without templates (i.e., using a Gaussian blob). Picked particles were extracted into boxes of 256 × 256 pixels. Initial 2D classification was carried out in Cryosparc (Punjani et al., 2017) in order to remove junk particles. All further processing was performed in RELION 2.1 (Scheres, 2012). For both datasets, the remaining particles were subject to a consensus 3D auto-refinement procedure using the RPc (EMD-3695) as an initial reference model (filtered to 60 Å). 3D classification was then performed without alignment to separate different complexes and/or conformational states. Classes for refinement were picked based on the presence of DNA. The 3D class corresponding to RPip model underwent a further round of 3D classification with the best class (based on quality of DNA density) chosen for further processing. Individual, homogeneous classes were then re-refined and post-processed (masked and sharpened) resulting in the final maps to resolutions of 3.4 Å (RPo), 3.7 Å (RPitc) and 4.1 Å (RPip), according to the gold-standard Fourier shell correlation (FSC) at 0.143 criterion.

Masks for postprocessing were generated within RELION with an initial extension of 2 pixels and a soft edge of 3 pixels. Additionally, maps filtered to local resolution values were generated from RELION and were subsequently used for model building.

Although initially RPip conformation was not observed within the RPo dataset during data processing described above, multiple additional rounds of classification within Cryosparc (28) revealed a small subset (5%) of the data corresponding to this complex. This conformation was not easily detected in the RPo dataset, possibly due to the smaller size of the RPo dataset (∼1/3 of that in RPitc dataset) and the differences in particle orientations. Due to the small number of these particles from RPo dataset and the different data parameters, we did not include these particles in the final RPip reconstruction.

#### Model building, refinement and structural analysis

The RNAP-σ^54^ closed complex (PDB: 5NSR) was used as an initial model for the building of the RPitc structure in Coot (Emsley et al., 2010). Protein subunits were manually adjusted into the density and the transcription bubble was built using an ideal B-form dsDNA at the upstream and downstream edges of the bubble with ssDNA built into the density to connect the two edges. This model was used as the initial model for RPo, RPitc and RPip. For RPo and RPitc, only minor manual adjustment of the structures were required to fit the structure into the density. For RPip β’ subunit was subject to flexible fitting using MDFF (Trabuco et al., 2009) while the DNA was built by distorting an ideal B-form dsDNA. All models were subject to jelly body refinement using Refmac (Murshudov et al., 2011) and real space refinement in Phenix (Afonine et al., 2012).

All the structural comparisons and analysis were performed in PyMol (DeLano, 2002) and Chimera (Goddard et al., 2007). All figures and morph movies are made in PyMol and Chimera.

#### Small primed (sp) RNA assays

On super-coiled *nifH* promoter template DNA using [α-^32^P] GTP radiolabelled nucleotides, reactions were performed in 10 ul final volumes containing: 100 nM holoenzyme (1:4 ratio of RNAP: σ^54^) and 20 nM promoter DNA probe, 4 μM PspF1-275 and 4 mM dATP in STA buffer (Burrows et al., 2010) was incubated at 37°C for 20 min before synthesis of sp RNA. Synthesis was initiated by adding 0.5 mM dinucleotide primer UpG, 0.2 mCi/ml [α-^32^P] GTP (3000 Ci/mmol) and 0.2 mg/ml heparin. After incubation at 37°C for 10 min, the reaction mixtures were quenched by addition of 4 μl of denaturing loading buffer and run on a 20% denaturing gel and visualized using a Fuji FLA-5000 Phosphorimager.

On super-coiled *nifH* promoter template using cold GTP nucleotide, reactions were performed as above, but instead of using the [α-^32^P] GTP, 1 mM GTP was added. Sp RNA samples were then labeled by by [γ-^32^P] ATP.

On pre-opened (−10 to −1) linear DNA probe, reactions were performed in 10 mM Tris (pH8.0), 150 mM NaCl and 10 mM MgCl_2_ by adding 8.5 μM RNAP-σ^54^_R336A_, 12.8 μM DNA, 1 mM GTP and 1 mM UpG and after incubation at 4°C for 1 hour, spRNA samples were then labeled by [γ-^32^P] ATP. The reaction mixtures were quenched by addition of loading buffer and run on a 20% denaturing gel and RNA products were visualized after the reaction (see below) using a Fuji FLA-5000 Phosphorimager.

#### DNase I and RNase T1

Where appropriate, either 1 U (final) DNase I (Roche) or 10 U (final) RNase TI (Fermentas) was added to the spRNA reaction (after spRNA synthesis) and the reaction was incubated at 37°C for 10-60 min to initiate cleavage. The reaction was quenched by addition of denaturing loading buffer and the reactions were analyzed as described above.

#### Phosphorylation reactions for labeling RNA with [γ-^32^P] ATP

Where appropriate the spRNA was phosphorylated at 37°C for 30 min using 1 U (final) T4 PNK, 1 μL of 10 × reaction buffer, and 0.3 mCi/ml [γ-^32^P] ATP (3000 Ci/mmol). The reaction was quenched by addition of denaturing loading buffer and the reactions were analyzed as described above.

#### RNA sequence analysis

The products of the transcription reaction performed at 4°C with 1mM GTP and 1 mM UpG using RNAP-σ^54^R336A and a pre-opened linear DNA probe, as described in the small primed RNA assays section, were analyzed as follows: a poly(A) tail was added to the 3‘ terminus of the RNAs using *E. coli* Poly(A) Polymerase (5 units, New England Biolabs) in the presence of 1 mM ATP. The 10 μL reaction was incubated at 37°C for 1 hour. Then an RNA adaptor with a 3′ phosphate (5′-P-ACUCCGAUAUCACGCUU-P-3′) was ligated to the 5′ terminus of the RNAs using RtcB RNA Ligase (15 pmol, New England Biolabs) in the presence of 0.1 mM GTP and 1 mM MnCl_2_. The 20 μL reaction was incubated at 37°C for 1 hour. Subsequently, cDNA was synthesized in a 100 μL reaction using an oligo(dT) primer (5′-GGGAGGCCCCTTTTTTTTTTTTTTTT-3′) and SuperScript III Reverse Transcriptase (ThermoFisher Scientific) and 1/10 of the cDNA synthesis reaction volume was added in a PCR using GoTaq® Green Master Mix (Promega), the oligo(dT) primer and 5′-ACTCCGATATCACGCTT-3′. Following 2% (^w^/_v_) agarose electrophoresis the amplicons were extracted and purified using the MinElute Gel Extraction Kit (QIAGEN) and cloned using the pGEM-T Easy Vector System (Promega). The ligation reaction was transformed into XL10-Gold® Ultracompetent Cells and recombinant plasmids were identified using blue/white selection on Luria-Broth agar/amplicillin/IPTG/X-gal plates, purified using QIAprep® Spin Miniprep Kit (QIAGEN) and sequenced by GENEWIZ. During the bioinformatics analysis, the adaptor, the oligo(dT) primer and the poly(A) tail were correctly identified on the sequences and the original RNA was determined as UGGG, located between the adaptor and the poly(A) tail. All protocols were performed according to the instructions of the manufacturers unless otherwise indicated above.

### Data and Software Availability

The accession numbers for the cryoEM reconstructions and the corresponding structural models reported in this paper are EMD-0002, PDB: 6GH6 (RPip), EMD-0001, PDB: 6GH5 (RPo), EMD-4392, and PDB: 6GFW (RPitc).

## Supplemental Information

Document S1. Figures S1–S7

Document S2. Article plus Supplemental Information
