# Supplementary material for: Structures of Bacterial RNA Polymerase Complexes Reveal the Mechanism of DNA Loading and Transcription Initiation
Source: Mol Cell. 2018 Jun 21;70(6):1111–1120.e3. doi: 10.1016/j.molcel.2018.05.021 (PMC6028918; doi:10.1016/j.molcel.2018.05.021)
Supplement: Document S1. Figures S1–S7 [file mmc1.pdf]

Molecular Cell, Volume *70*

Supplemental Information

Structures of Bacterial RNA Polymerase Complexes Reveal the Mechanism of DNA Loading and Transcription Initiation

Robert Glyde, Fuzhou Ye, Milija Jovanovic, Ioly Kotta-Loizou, Martin Buck, and Xiaodong Zhang

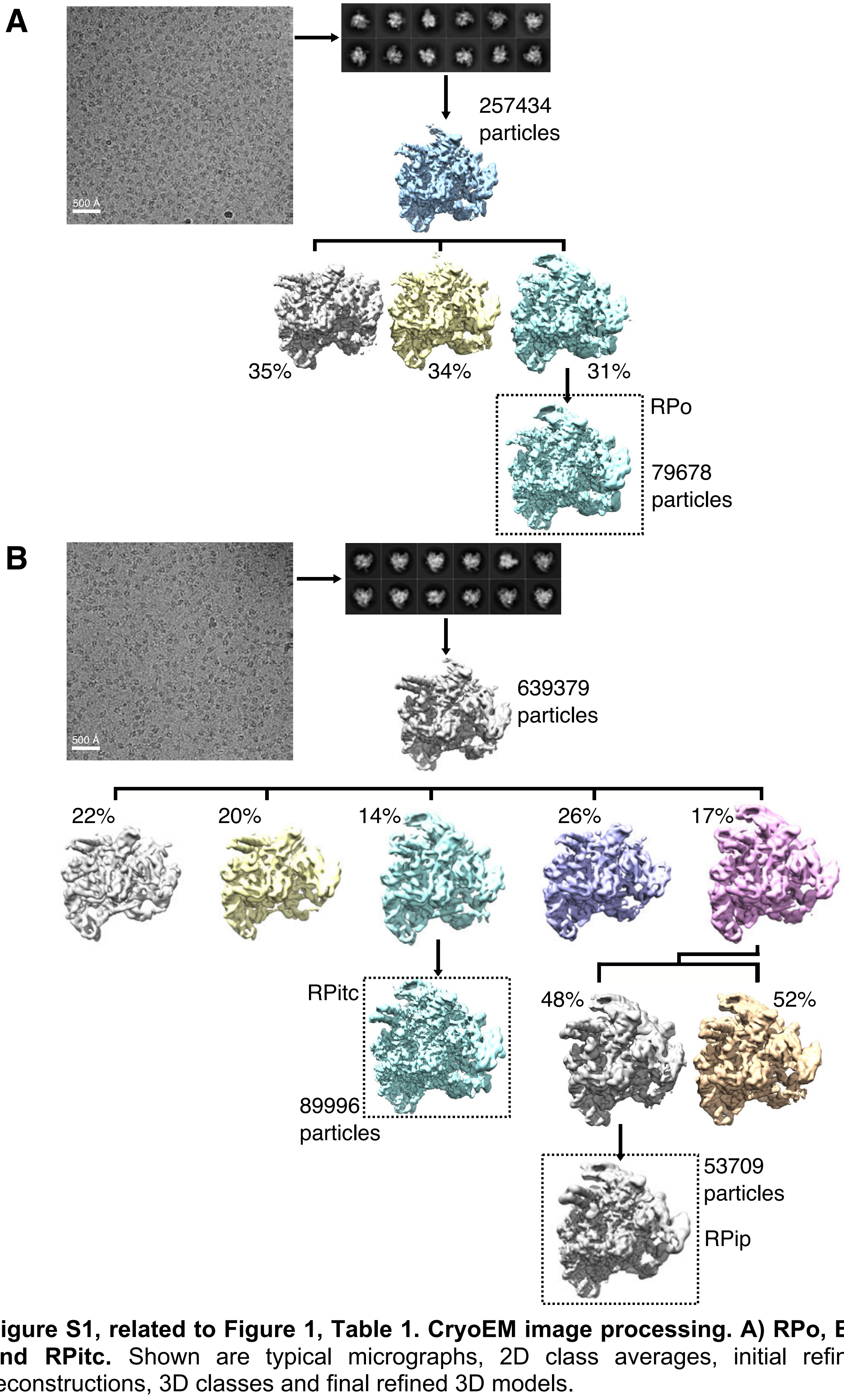

Figure S1, related to Figure 1, Table 1. CryoEM image processing. A) RPo, B) RPip and RPitc. Shown are typical micrographs, 2D class averages, initial refined 3D reconstructions, 3D classes and final refined 3D models.

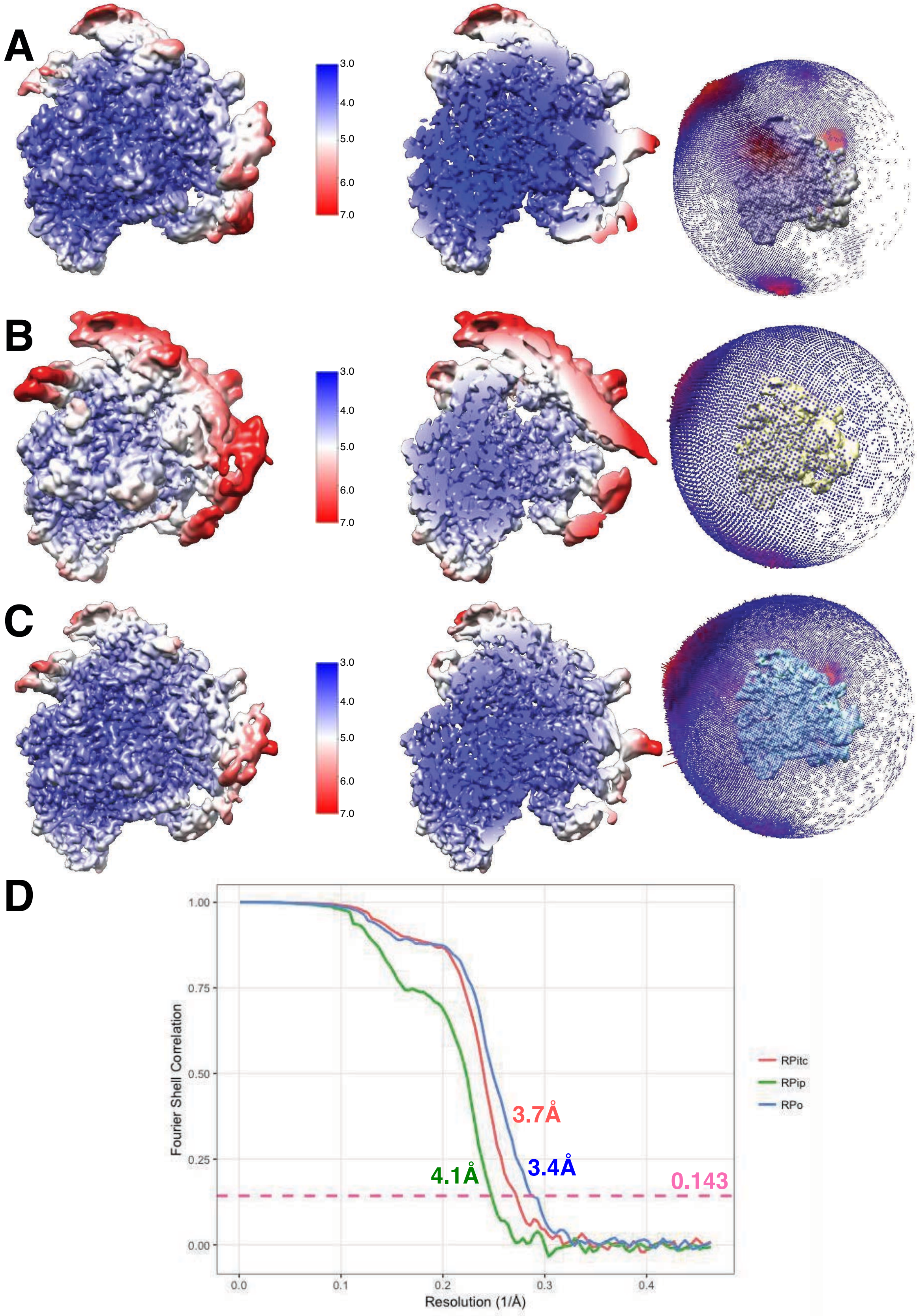

Figure S2, related to Figure 1 and Table1. Quality of the reconstructions. A) RPo, B) RPip and C) RPitc. Shown are local resolution map on surface and through the centre sections, angular distribution of all the particles used in final reconstructions. **D**) Fourier

shell correlation coefficients (FSC) of two half maps and the resolution based on 0.143 criteria.

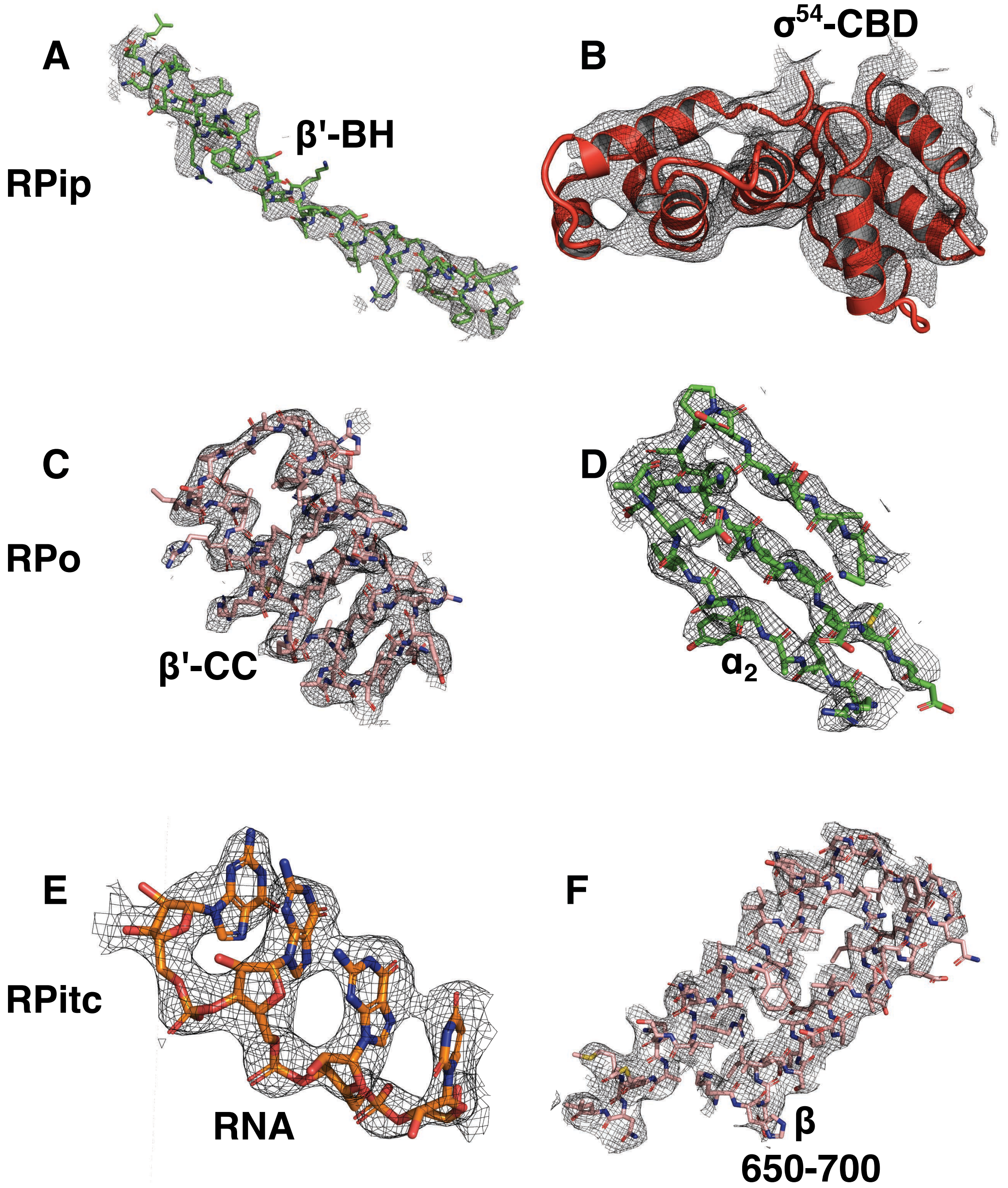

Figure S3, related to Figure 1 and Table 1. Example electron density map of representative regions. A)-B) RPip, C)-D) RPo and E)-F) RPitc. Density for synthesized RNA can be clearly seen **(E)**.

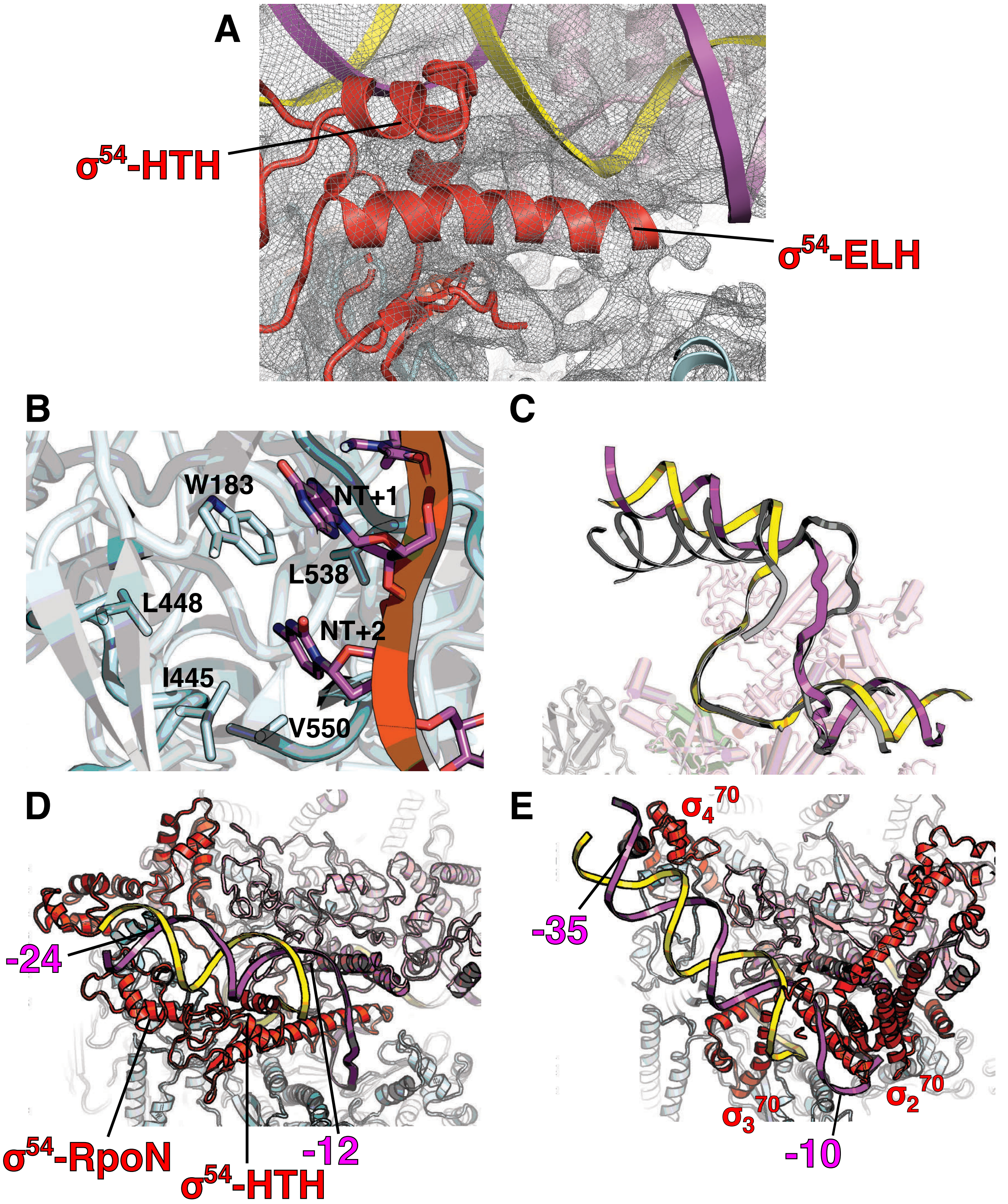

Figure S4, related to Figure 2 and Figure 3. Transcription bubble stabilisation in RNAP-σ^{54} RPo/RPitc and comparisons with RNAP-σ^{70} RPo/RPitc. A) Electron density suggests alternative path for ELH in RPip compared to those in RPc or RPo and RPitc. **B)** Downstream ss-dsDNA junction in RPo. +2 NT base is inserted into a hydrophobic pocket and +1 NT base forms hydrophobic-base interactions with W183 of β-subunit. **C**) Comparison of transcription bubble in RPitc (yellow – T strand, magenta – NT strand) with those of RNAP-σ^{70} RPitc (4YLO, grey). **D)** Promoter binding in RNAP-σ^{54} open complex, **E**) same view as in *D)* but with RNAP-σ^{70} open complex.

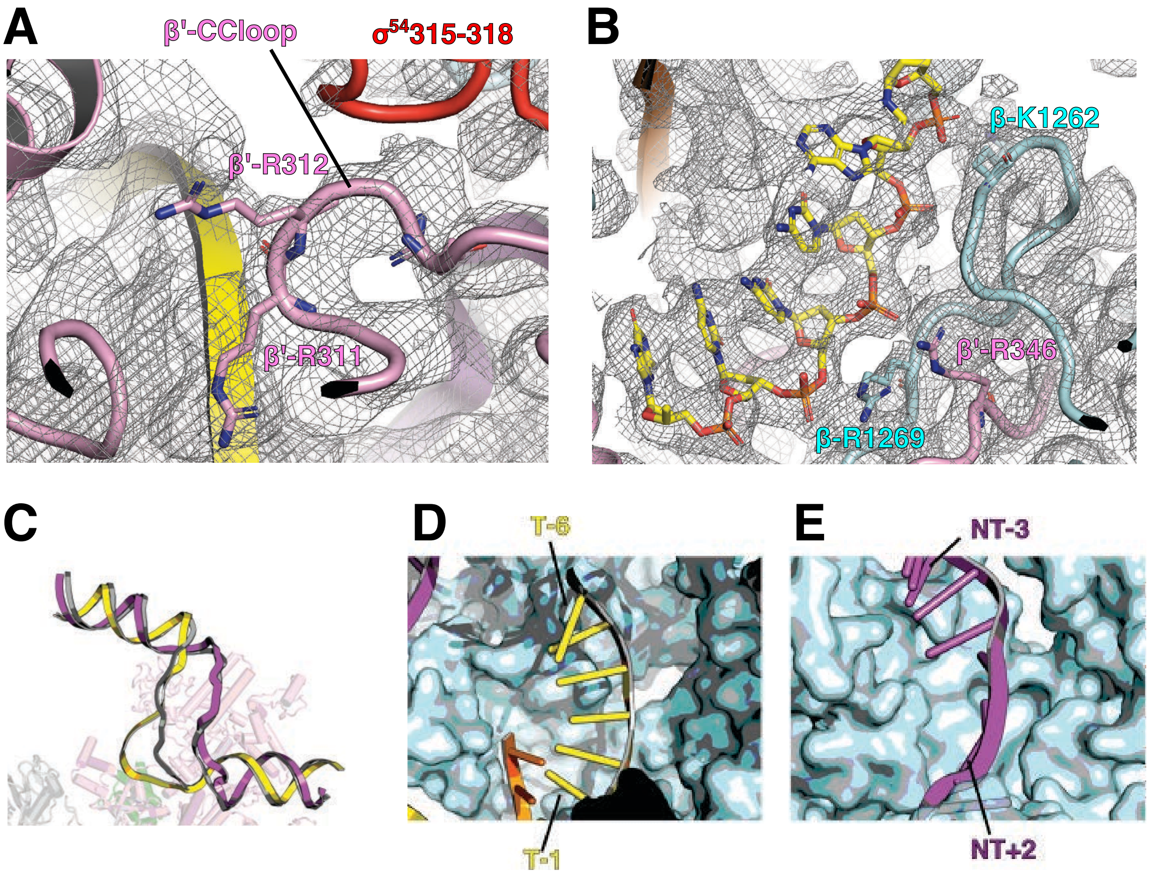

Figure S5, related to Figure 4. DNA in *de novo* synthesizing initial transcribing complex (RPitc). A). Role of the β' coiled coil loop in interacting with T-strand **B)** Template strand is further stabilised by positively charged residues in β an β' subunits. **C)**. Comparisons of the transcription bubble in RPo (grey) and RPitc (yellow and magenta) showing that the template strand is expanded in RPitc, **D**) Cavity behind T strand that would accommodate the scrunched up DNA. E) Cavity behind NT strand.

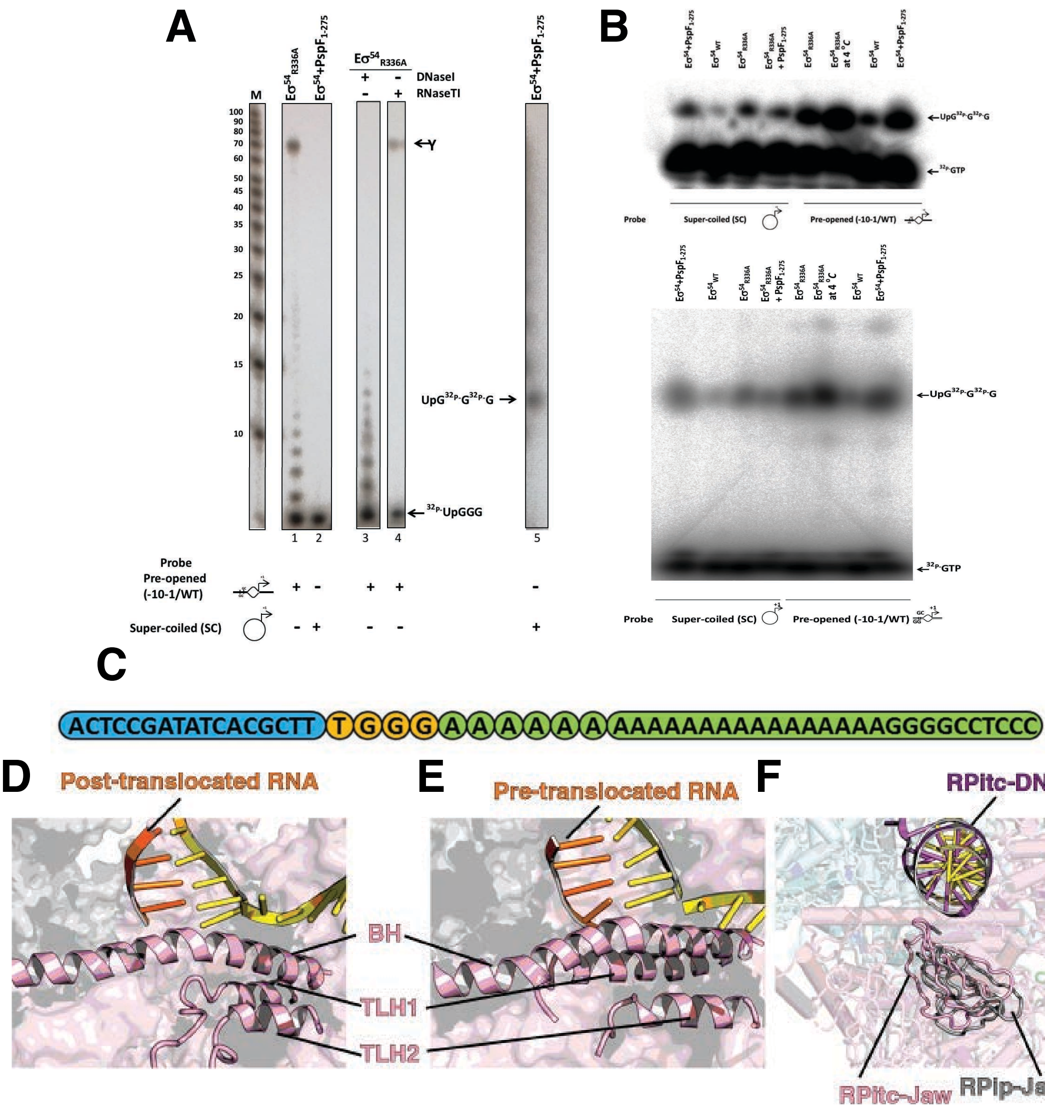

Figure S6, related to Figure 4. *De novo* RNA synthesis and comparison with that of RNAP-σ^{70}. A) sp RNA produced from super-coiled (SC) *nifH* promoter template DNA and the pre-opened (-10-1/WT) linear DNA probes. sp RNA samples were labelled by kinasing, using the [γ-^{32}P] ATP. Denaturing gel showing: lane 1- spRNA produced from RNAP (E)-σ^{54}_{R336A} at 4 ^{o}C from pre-opened template, lane 2- spRNA produced from SC DNA in the presence of WT Eσ and PspF activator at 37 ^{o}C. A further control demonstrate that slower-migrating product (Y) is sensitive to DNaseI (lane 3) and spRNA is sensitive to RNaseT1 (lane 4) (*1*), lane5- spRNA produced from SC DNA in the presence of WT Eσ, PspF activator, and the [α-^{32}P] GTP at 37 ^{o}C with a 5'OH migrated slower than a marker's (M) 10 base long RNA band. **B)**. Synthesis of the spRNA UpG^{32p}G^{32p}G. WT RNAP-σ^{54} was used as negative control. Upper panel - short 20% denaturing gel, lower panel - long 20% denaturing gel. **C)**. Exemplary result following RT-PCR, cloning and sequencing of the major RNA product (orange) of the transcription reaction, which is located between the adapter (blue) and the poly(A) tail/oligo(dT) primer (green). **D**) In RPitc, RNA-DNA structure in post-translocation position, trigger loop/helix in open conformation. **E)** RNAP-σ^{70} RPitc, RNA-DNA structure in pre-translocation position, trigger loop/helix in closed conformation, **F)** β'-jaw domain comparisons between RNAP-σ^{54} and σ^{70} RPitcs.

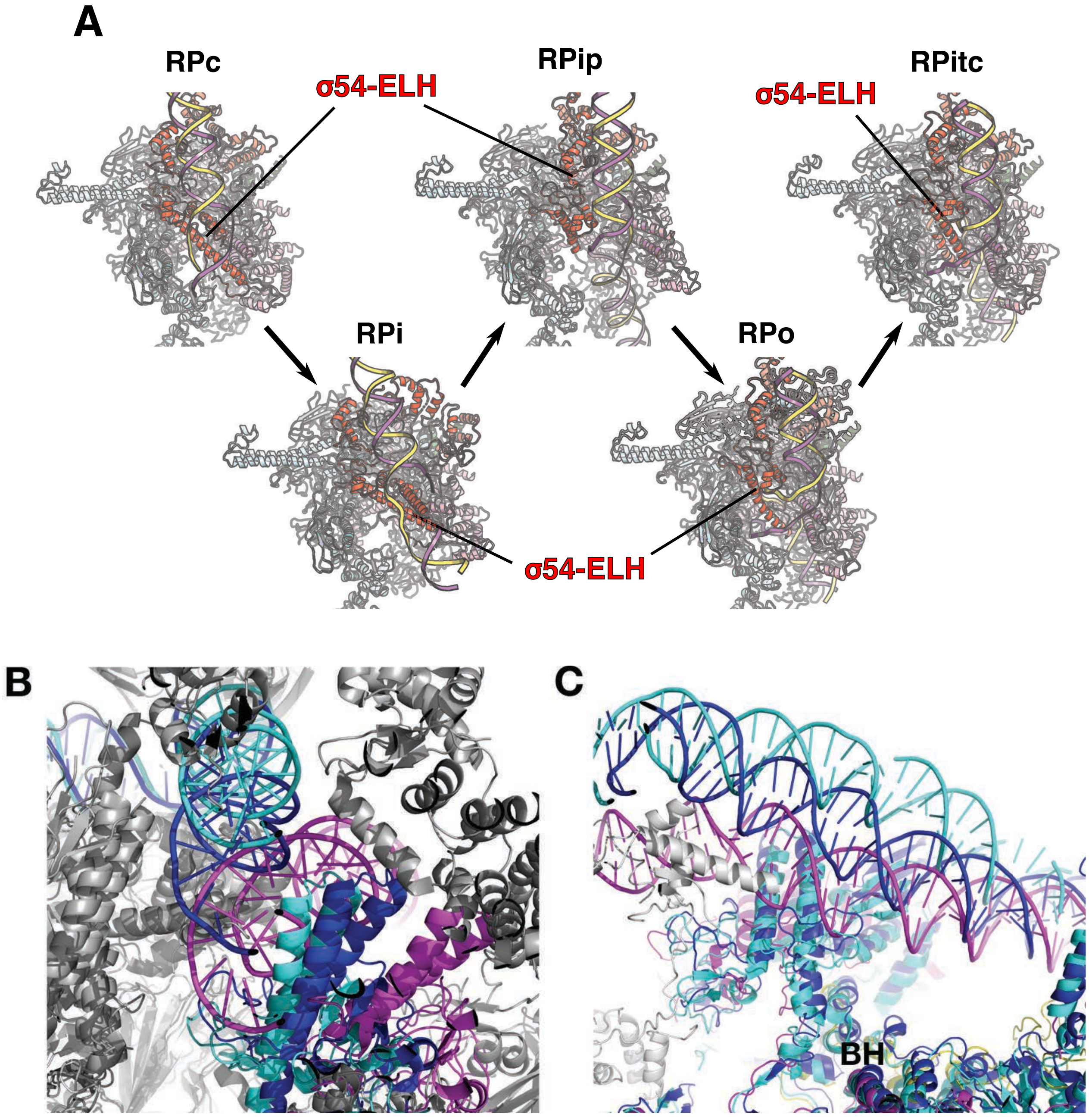

Figure S7, related to Figure 5 and Figure 6. Coordinated movements during transcription initiation. A) Coordinated movements of ELH, RNAP clamps and DNA during isomerisation in RNAP-σ^{54} system. **B)** Comparisons of RPip (magenta) with yeast Pol II CC (pdbcode 5FZ5, cyan) and human Pol II CC (pdbcode 5IYA, blue) showing the clamp opening is correlated with DNA positioning. C). Same as in B) but viewed from β side. Structures are aligned on the bridge helix (BH). DNA and clamp are colored while other subunits are shown in grey.
